# *Clostridium chauvoei*, an Evolutionary Dead-End Pathogen

**DOI:** 10.3389/fmicb.2017.01054

**Published:** 2017-06-09

**Authors:** Lorenz Rychener, Saria In-Albon, Steven P. Djordjevic, Piklu Roy Chowdhury, Pamela Nicholson, Rosangela E. Ziech, Agueda C. de Vargas, Joachim Frey, Laurent Falquet

**Affiliations:** ^1^Institute of Veterinary Bacteriology, Vetsuisse Faculty, University of Bern Bern, Switzerland; ^2^The iThree Institute, University of Technology Sydney, Ultimo NSW, Australia; ^3^Department of Preventive Veterinary Medicine, Federal University of Santa Maria Santa Maria, Brazil; ^4^Department of Biology, Swiss Institute of Bioinformatics, University of Fribourg Fribourg, Switzerland

**Keywords:** *Clostridium chauvoei*, CRISPR, virulence genes, flagellin genes, blackleg, dead-end evolution

## Abstract

Full genome sequences of 20 strains of *Clostridium chauvoei*, the etiological agent of blackleg of cattle and sheep, isolated from four different continents over a period of 64 years (1951–2015) were determined and analyzed. The study reveals that the genome of the species *C. chauvoei* is highly homogeneous compared to the closely related species *C. perfringens*, a widespread pathogen that affects human and many animal species. Analysis of the CRISPR locus is sufficient to differentiate most *C. chauvoei* strains and is the most heterogenous region in the genome, containing in total 187 different spacer elements that are distributed as 30 – 77 copies in the various strains. Some genetic differences are found in the 3 allelic variants of *fliC1*, *fliC2* and *fliC3* genes that encode structural flagellin proteins, and certain strains do only contain one or two alleles. However, the major virulence genes including the highly toxic *C.chauvoei toxin A*, the sialidase and the two hyaluronidases are fully conserved as are the metabolic and structural genes of *C. chauvoei*. These data indicate that *C. chauvoei* is a strict ruminant-associated pathogen that has reached a dead end in its evolution.

## Introduction

*Clostridium chauvoei*, a Gram positive, highly pathogenic, strict anaerobic bacterium that is able to sporulate, is the etiologic agent of blackleg, a severe disease specifically of cattle and to a lesser extent also of small ruminants. Blackleg spreads globally manifesting as a fulminant myonecrosis that generally leads to the death of the animal within a short time. Due to the high mortality blackleg causes significant losses in livestock production (Hatheway, 1990; Groseth et al., 2011; Frey and Falquet, 2015). Pathological lesions of blackleg are mostly found in the muscular tissue of animals, often in leg muscles, from where the pathogen is commonly isolated. Animals generally get infected from *C. chauvoei* spores that contaminate the soil of pastures either from perished animals or via manure (Bagge et al., 2009; Lange et al., 2010). In cattle, the pathogen is taken up via the digestive tract or the respiratory tract from where *C. chauvoei* migrates to the muscle tissues where the spores remain dormant until specific conditions are generated, as tissular devitalization that promotes anaerobiosis, resulting in their germination, multiplication and consequently production of the exotoxins (Jubb et al., 1991). In small ruminants, skin lesions are also considered as a port of entry of the pathogen. Blackleg occurs with increased incidence during dry seasons that followed flooding, when the animals have to graze short plants and are closer in contact with their nostrils and muzzles to soil where the pathogen spreads during the preceding flooding (Hatheway, 1990; Useh et al., 2006a). *C. chauvoei* is considered as one of the most pathogenic *Clostridium* species. The infection by *C. chauvoei* causes myonecrosis, oedemic lesions and fever that is rapidly followed by lameness and death. Due to the rapid death of the animals, which mostly is the only sign of the disease, antibiotics are generally not used to cure blackleg. However, commercial and locally produced vaccines successfully control blackleg in ruminants (Uzal, 2012; Frey and Falquet, 2015). Blackleg is not notified as a zoonotic disease, although two isolated cases of human infections with *C. chauvoei* leading to lethal fulminant gas gangrene and neutropenic enterocolitis have been reported (Nagano et al., 2008; Weatherhead and Tweardy, 2012).

Current knowledge on pathogenicity of *C. chauvoei* reveals that toxins, a highly active DNAse, hyaluronidase, sialidase, and flagella represent the main virulence factors (Useh et al., 2003, 2006b; Vilei et al., 2011; Frey et al., 2012; Frey and Falquet, 2015). Among the postulated toxins, *C.chauvoei* toxin A (CctA), a member of the β-barrel pore forming leukocidin superfamily, was shown to represent the main cytotoxic- and haemolytic activity of *C. chauvoei*. Recombinant purified CctA toxin produced in *Escherichia coli* is oxygen stable with regard to its haemolytic and cytotoxic activity. Guinea pigs vaccinated with a purified inactivated recombinant chimeric protein consisting of the CctA toxin and the *E. coli* heat labile toxin subunit B (LTB) as an adjuvant were protected against challenge with virulent *C. chauvoei* spores, revealing the central role of CctA in virulence (Frey et al., 2012). Besides toxin CctA, two further virulence factors, sialidase NanA and hyaluronidase NagH have been analyzed biochemically and genetically. The enzymes seem to enable *C. chauvoei* to stir from the initial site of infection which are generally believed to be oral or the respiratory tract or occasionally skin lesions, and get access to muscular tissue where the bacterium can replicate and cause myonecrosis, the typical pathological symptoms of blackleg (Vilei et al., 2011; Frey and Falquet, 2015).

Vaccines against blackleg consist of chemically inactivated bacteria of mostly relatively ancient strains providing outer membrane proteins and flagellar proteins that have been proposed as immunogens and bacterial culture supernatants that are expected to contain the main toxins (Mattar et al., 2002; Uzal, 2012). Recently it has been shown that the CctA toxin alone, which is conserved between *C. chauvoei* strains isolated worldwide, prepared by recombinant gene technology, provided full protection in a guinea pig infection model which serves as biological test for potency tests in the batch release procedure of commercial vaccines (Frey et al., 2012). Flagellar antigens are traditionally considered to be important virulence attributes and protective factors in vaccines (Chandler and Gulasekharam, 1974; Tamura and Tanaka, 1987; Tanaka et al., 1987; Tamura et al., 1995). They have been used recently as diagnostic tools for the serological detection of *C. chauvoei* infections and were shown to be antigenically highly conserved between the type strain ATCC10092 and strains isolated in India between 2010 and 2012 (Usharani et al., 2015).

Due to the short duration of infection of the bacterium in the animal host, during which the bacterium replicates extensively and forms spores that can survive for several years on pastures, *C. chauvoei* has a bi-modal lifestyle, similar to *B. anthracis*. Although it is generally known that pastures contaminated with spores of *C. chauvoei* represent the main epidemiological hazard for infection of cattle (Wolf et al., 2017), and that vaccination is the most efficient way to stop the epidemic, there is no knowledge of the phylogeny or molecular epidemiology of *C. chauvoei*.

In order to get insight into the molecular genetic evolution of *C. chauvoei*, to provide new tools for molecular epidemiological assessments, and to provide a scientific basis for the development of both novel vaccines as well as potency test that is independent of live animal infections, we have fully sequenced, analyzed and compared the genomes of 20 strains of *C. chauvoei* that were isolated between 1951 and 2015 and originated from Africa, Australia, Northern and Southern America and Europe. These strains originate mostly from internal strain repositories as there are only very few strains at public collections. Particular focus was given to the genetic variability of known and potential virulence genes such as the flagellar genes that provide motility.

## Materials and Methods

### Bacterial Strains

Bacterial strains used in this study are listed in **Table 1**. They were sourced from a collection maintained at the Institute of Veterinary Bacteriology, University of Bern and originate either from own isolations from local blackleg cases, or were obtained during the last decades from various institutions worldwide for the purpose of reference species identifications or were ordered from international strain collections.

**Table 1:** List of all strains included in the study with strain name, year of isolation, location of isolation, host animal, strain collection, reference and SNVs detected during the analysis.

Strain nr.	Year of isolation	Origin	Animal host	Original strain collection / nr	Reference	SNVs	SRA Accession
JF1863	Unknown	Unknown	Bovine	ATCC10092^T^ Type strain		134	SRR5429447
JF2696	2002	Brazil	Bovine	Vetsuisse University Bern		295	SRR5429446
JF2697	2002	Brazil	Bovine	Vetsuisse University Bern		165	SRR5429445
JF2698	2002	Brazil/unknown	Bovine	Vetsuisse University Bern		–	–
JF3703	1956	New Zealand	Ovine	Wellcome Collection Cf 7		7797	SRR5429444
JF3837	1965	Switzerland	Bovine	Vetsuisse University Bern	University Bern, Thesis J. Martig	75	SRR5429443
JF3838	1965	Switzerland	Bovine	Vetsuisse University Bern	University Bern, Thesis J. Martig	105	SRR5429442
JF3840	1965	Switzerland	Bovine	Vetsuisse University Bern	University Bern, Thesis J. Martig	117	SRR5429441
JF3841	1965	Switzerland	Bovine	Vetsuisse University Bern	University Bern, Thesis J. Martig	131	SRR5429440
JF4057	2007	Switzerland	Bovine	Vetsuisse University Bern		101	SRR5429439
JF4251	2002	Sweden	Bovine	SLU Uppsala	Vilei et al., 2011	95	SRR5429438
JF4252	2006	Sweden	Bovine	SLU Uppsala	Vilei et al., 2011	145	SRR5429437
JF4253	2007	Sweden	Bovine	SLU Uppsala	Vilei et al., 2011	141	SRR5429436
JF4335	2004	Switzerland	Bovine	Vetsuisse University Bern	Falquet et al., 2013	34	SRR5429435
JF4491	1956	New Zealand	Ovine	Wellcome collection Cf 7		8495	SRR5429434
JF4492	1959	United States	Bovine	Vetsuisse University Bern		244	SRR5429433
JF4493	1959	Australia	Bovine	Wellcome collection Cf 84		7991	SRR5429432
JF4494	1963	New Zealand	Unknown	Wellcome collection Cf 1		8515	SRR5429431
JF4495	1953	United Kingdom	Bovine	Wellcome collection Cf44	Frey et al., 2012	8520	SRR5429430
JF5504	2013	Switzerland	Bovine	Vetsuisse University Bern		–	–
JF5806	1951	South Africa	Ovine	NCTC 8361		248	SRR5429429
JF5842	2015	Brazil	Bovine	UFSM Jaguari SB 43/15	R. Ziech, UFSM Brazil	272	SRR5429428


**FIGURE 1:**
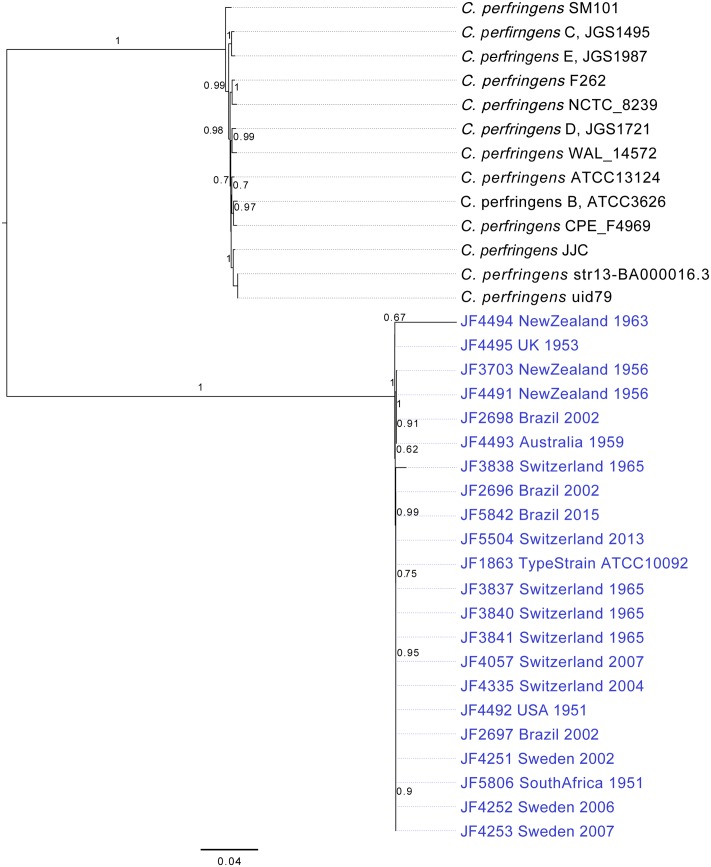
Phylogenetic Trees of the 20 *C. chauvoei* strains; tree generated with the alignment of concatenated sequence of 37 marker genes searched from the genome of *C. chauvoei* and *C. perfringens* strains whose full genome sequences are publicly accessible, using the PhyloSift software. The bar in the figure indicates substitutions per site. Confidence values are given at the major branching points.

*Note that strains JF2698 and JF5504 were only used for **Figure 1**.*

### Update of Reference Strain JF4335

The genome of *C. chauvoei* strain JF4335 was sequenced with Pacific Bioscience RSII. After circularization, the sequence was corrected with Illumina HiSeq reads for the substitutions due to sequencing errors and for the frameshifts. The resulting full genome was annotated using the Prokka pipeline (Seemann, 2014) using annotation from the previous draft genome JF4335 (GenBank: CBML000000000.1) (Falquet et al., 2013) and *C. perfringens* ATCC 13124 (Genbank: NC_008261.1) and UniProtKB as additional database.

### Whole Genome Sequencing and SNVs Discovery

The DNAs of the 20 *C. chauvoei* strains were subjected to sequencing by Illumina HiSeq using the Nextera XT DNA protocol for the library preparation and the V3 chemistry to produce 2 x 250 bp paired-end reads (Fasteris SA, Plan-les-Ouates, Switzerland). The number of reads after filtering ranges from 0.6 to 4 million paired-end reads. All reads are available on the Sequence Read Archive (SRA: SRR5429428 – SRR5429447) and strain number and SRA accession number are linked in **Table 1**. The full genome of *C. chauvoei* strain JF4335 (LT799839), obtained as described above, was used as a reference. Illumina paired-end reads from all strains were mapped to the reference using Bowtie2 v2.2.4 with default settings (Langmead et al., 2009). The variant calling was performed for each alignment using the Samtools package v1.3 (Li et al., 2009). All SNVs in the VCF tabular files were concatenated into a FASTA alignment^1^. As a control group Illumina HiSeq 2500 reads from *C. septicum* (SRA: DRP001713) were used and submitted to the same procedure.

In a previous work a cryptic plasmid was identified (Frey and Falquet, 2015). To find this cryptic plasmid the contigs from the *de novo* assembly were mapped against the reference PacBio sequence of JF4335 using Bowtie2 v2.2.4 with default settings. Additionally, the raw reads of all 20 strains were assembled with plasmidSPAdes (Antipov et al., 2016).

### Phylogenetic Analysis

The genomes were assembled at the ithree institute (University of Technology Sydney, Australia) using an in-house bioinformatics genome assembly and analysis pipeline (Darling et al., 2014a). Briefly, genomes were assembled using the A5-miseq pipeline (Tritt et al., 2012; Coil et al., 2015), which can process reads up to 500nt long and constructs de Bruijn graphs with **k**-mers up to 500 nt.

The initial phylogenetic tree was constructed using PhyloSift (Darling et al., 2014b). PhyloSift works by identifying homologs of universally conserved 37 single copy elite markers that are not dependent on operational taxonomic units (OTU; which account for approximately 1% of an *Escherichia coli* genome) from any given draft bacterial genome. These include 30 ribosomal protein genes and 7 genes encoding proteins of the translation initiation, elongation and termination process (Darling et al., 2014b). For the purposes of this study, PhyloSift was used to create a concatenated multiple alignment of the marker genes from all our draft genomes. Thirteen *C. perfringens* genomes from GenBank were included as outgroups. From this alignment, a phylogeny was inferred using FastTree2 (Price et al., 2010) and the resulting tree including the confidence scores (Darling et al., 2014b) was visualized using FigTree v1.4.0^2^ (**Figure 1**). Internal tree branches were annotated with the support value for each of the clades.

In addition to this explorative approach, all strains were checked for the presence of known genes involved in pathogenicity. Specifically, the putative coding regions for the gene encoding the cytotoxin CctA, hyaluronidases NagH and NagI, and sialidase NanA were analyzed in detail by mapping the respective gene to the *de novo* contigs of each strain and by that extracting the corresponding open reading-frame (ORF) from the strains. To assess sequence similarity, we performed a multi-sequence alignment with the translations of the extracted ORFs using MAFFT v7.222 (Katoh et al., 2002; Scoring Matrix: 200PAM/*k* = 2).

### Analysis of Single Nucleotide Variations (SNV)

Based on the FASTA alignment of the SNVs a phylogenetic tree was created using the RAxML (options: -m GTRCAT -f d -N 10′000 -d) (Stamatakis, 2014). Bootstrap values were calculated using RAxML using 10’000 simulations. The tree was visualized and rooted at the outgroup *C. septicum* with FigTree v1.4.3^3^. In addition to the phylogenetic tree, a cladogram was created in FigTree.

### CRISPR Extraction and Comparison

To predict the Clustered Regularly Interspaced Short Palindromic Repeats (CRISPR) sequences, the Illumina reads were *de novo* assembled using SPAdes v3.9.0 (Nurk et al., 2013). The resulting contigs were uploaded to CRISPRFinder (Grissa et al., 2007) for CRISPR prediction. The spacer sequences were oriented within the contigs of each strain based on the CRISPR-associated endoribonuclease 2 (*cas2*) gene of the reference strain JF4335. Spacer sequences were compared, within and between strains, with each other to find overlapping or unique spacer sequences. They were furthermore compared, within and between strains. Each different spacer sequence was defined as unique, since it was shown that a single nucleotide change in a spacer sequence alters its specificity (Barrangou et al., 2007). For this step NUCmer v3.06 (Kurtz et al., 2004; options: –maxmatch -c 18 -g 3 -l 10) was used.

## Results

### Full Genome Analysis of *C. chauvoei* Reference Strain JF4335

*Clostridium chauvoei* strain JF4335, an isolate from a severe case of blackleg in Switzerland in 2004 was used as reference strain and as reference genome sequence for the species. The genome of this strain was sequenced using Pacific Biosciences R.S. (PacBio) sequencing (85,000 reads/2950 bp average read length). The reads of the PacBio sequencing were assembled with the SMRT Pipeline HGAP3 into one circularized closed contig. The chromosome sequence of *C. chauvoei* strain JF4335 is 2,887,451 bp with a G+C content of 28.3% and with a total of 2624 predicted coding sequences and 114 predicted structural RNAs including transfer tRNAs and ribosomal rRNAs. The complete, circularized and annotated genome was submitted to the European Nucleotide Archive (ENA) database (AC: LT799839). The PacBio sequence also revealed DNA methylations motifs of Restriction Methylation Systems (RMS): GTATA^m^C, CCAA^m^N_7_TTC/GAA^m^N_7_TTGG and GNGAA^m^AY where “A^m^” is the methylated base m6A. The first motif is a type II RMS (locus_tag = CCH01_18200), the second is a type I RMS (locus_tag = CCH01_22110) and the third was not identified.

### Phylogenetic Analysis of *C. chauvoei* Strains Collected World Wide

A thorough phylogenetic analysis of the 20 strains, based on a set of 37 OTU-free marker gene families with largely congruent phylogenetic histories revealed hardly any differences between the *C. chauvoei* strains originating from 4 different continents and collected over a period of 64 years. This in contrast to a sample of *C. perfringens* strains whose genomic sequences are published and which originate mostly from Europe and the United States isolated during the last few years (**Figure 1**). This indicates that *C. chauvoei* experienced virtually no genetic evolution in the observation period, compared to *C. perfringens*.

### Read Mapping and Consensus Comparison of the 20 International*C. chauvoei* Strains

**FIGURE 2:**
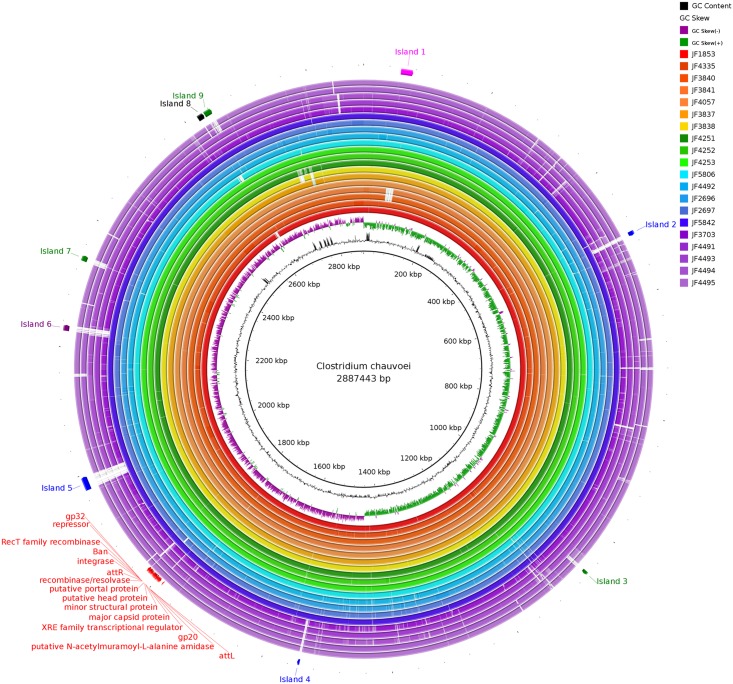
Consensus sequences of all strains aligned against the reference Pacific Biosciences sequence of JF4335 using BLAST Ring Image Generator. Most prevalent gaps (labeled as island 1–9) were inspected for genes potentially involved in virulence or pathogenicity. None of these genes were regarded as such. The gaps mostly represent fragments of transposases and fragments of phage DNA. In red the phages of JF4335, identified with PHASTER (Arndt et al., 2016), are indicated.

To get an overview of the genomes of the 20 *C. chauvoei* strains, the consensus sequences were extracted from the BAM files using SAMTools and visualized using the BLAST Ring Image Generator (BRIG) (Alikhan et al., 2011) (Upper Threshold 95%; Lower Threshold 90%) (**Figure 2**). A confirmation of the assumed, high similarity between the strains is apparent. The most prevalent gaps in **Figure 2** were identified and analyzed in more detail within the reference genome. The corresponding DNA segments of these gaps represented mostly fragments of transposases or fragments of putative bacteriophage genes. None of these gaps or islands contains any genes that would be known to have a vital function or to be involved in virulence or pathogenicity of the organism and therefore were not considered any further in this study.

**FIGURE 3:**
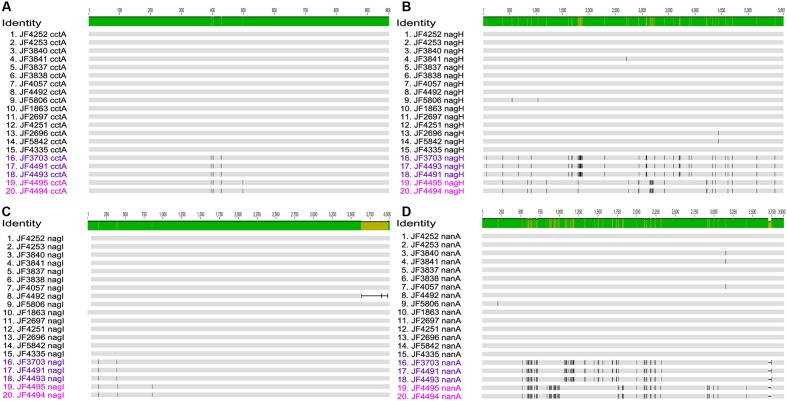
Multi-sequence alignment of the genes **(A)** *Clostridium chauvoei* toxin A (*cctA*), **(B)** Hyaluronidase (*nagH*), **(C)** Hyaluronidase (*nagI*), and **(D)** Sialidase (*nanA*) created with MAFFT v7.222 and visualized in Geneious v10.1.2. The number of SNV differences for each of these genes are given in the results section. Subgroups **(A,B)** of the Australian/New Zealand/United Kingdom cluster are differentiated by letters in dark and bright purple color, respectively.

The gene for the major virulence factor, cytotoxin CctA, was found to be present on the chromosome in all strains with an overall nucleotide identity of 99.6% (**Figure 3A**). In most strains, the *cctA* gene was identical to the reference strain JF4335. However, strains JF3703, JF4491 and JF4493 originating from New Zealand and Australia showed 3 single nucleotide variations (SNVs) representing silent mutations that lead to no difference in the amino acid (a.a.) sequences. Strains JF4494 (New Zealand) and JF4495 (United Kingdom) both revealed the four same SNVs of which one lead to a change in the a.a. sequence of the protein toxin changing the polar neutral glycine at position 167 to the basic a.a. arginine.

All strains contained two hyaluronidase genes *nagH* and *nagI* on the chromosome (**Figures 3B,C**) as found in the reference strain whereof *nagH* shows a pairwise identity of 99.7% (max. 42 SNVs out of 5661 bp) and gene *nagI* a pairwise identity of 99.1% (max. 4 SNVs out of 4005 bp) among the strains analyzed. The lower identity of *nagI* is mainly due to a 340 bp shorter ORF in strain JF4492. Apart from the slight shortening, the nucleotide sequences are almost identical. Minor differences were found in the strains from Australia/New Zealand/United Kingdom cluster, which revealed two SNVs in strains JF3703, JF4491 and JF4493, and three SNVs in JF4494 and JF4495. From these changes one leads to a change in a.a. valine to alanine at position 32 in all five strains and one in strains JF3703, JF4491 and JF4493 at position 115 changing the basic a.a histidine residue to the polar neutral tyrosine.

The gene for the predicted CDS of the sialidase (*nanA*) (**Figure 3D**) was found on the chromosome in all strains and showed a 99.0% pairwise identity (max. 47 SNVs out of 3900 pb). Most notable is a short deletion of 36 bases at the 3′-end of the nucleotide sequence of *nanA* in the strains from the Australian/New Zealand/United Kingdom cluster. Overall, the virulence genes encoding the main toxin CctA, hyaluronidases NagH and NagI and sialidase NanA are mostly identical or share a very high similarity in between strains. These similarities are even more pronounced in strains originating from the same country or continent. It is very apparent, that the group of strains originating from Australia, New Zealand and United Kingdom strikingly differs from the strains of the other European countries, Africa, and the Americas. Within the Australian/New Zealand/United Kingdom cluster, we see a more diverse pattern of SNVs, which permits to distinguish them further into subgroup A (JF3703, JF4491 and JF4493) and subgroup B (JF4494 and JF4995) as shown in **Figure 3**.

To investigate these different groups and attempting to differentiate those further, a single nucleotide variant (SNV) analysis and an analysis of the variable parts (spacers) of the CRISPR locus that was discovered within this study, was performed.

Using plasmidSPAdes analysis, we found a single circular contig of 4.1 kbp in all strains of the European/African/American cluster which corresponded to the same contig that did not get mapped to the chromosome of the reference strain JF4335. This circular contig, shares 95% identity with the cryptic plasmid previously described (Accession nr: HG323816.1) (Frey and Falquet, 2015). As previously noticed these plasmids contain only 4 very short ORF of 150 – 180 bp and contain no virulence genes.

### Single Nucleotide Variations (SNV) Analysis

**FIGURE 4:**
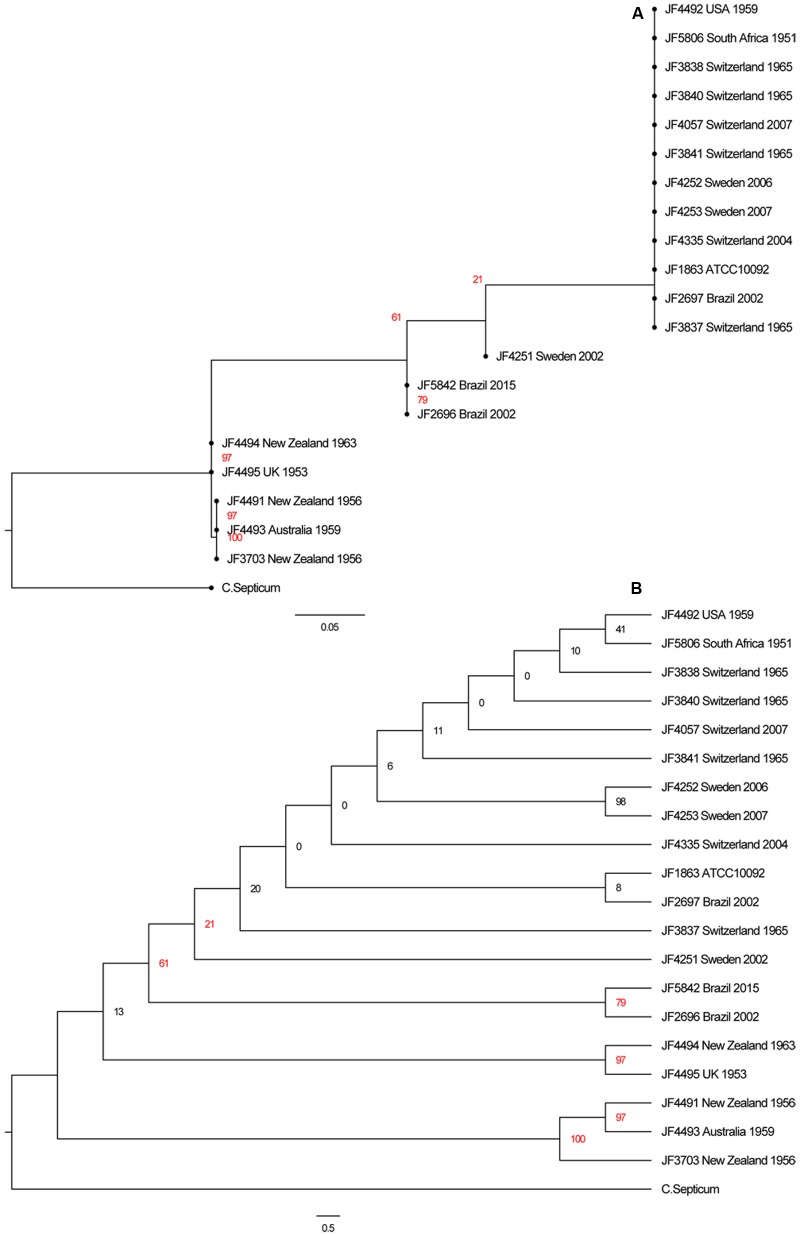
**(A)** Phylogenetic tree of the SNVs of all 20 *C. chauvoei* strains with a *C. septicum* strain as outgroup. **(B)** Cladogram of the same tree. Note that the bootstrap values of several branches are below 50% indicating that these branches are not well supported as this can be anticipated from the high genetic similarity between the individual strains of *C. chauvoei*.

In the phylogenetic analysis based on 37 OTU-free marker gene families we could identify virtually no difference between the different *C. chauvoei* strains. To create a better distinction between the twenty strains of *C. chauvoei*, we characterized them by whole genome SNV analysis. Paired-end sequencing was performed using the Illumina technology, yielding 0.6 and 4 million reads per strain. The filtered reads were mapped to the complete genome reference of *C. chauvoei* strain JF4335 obtained by PacBio sequencing. A total of 14,624 total chromosomal SNVs were identified among the worldwide collection of *C. chauvoei* strains. The numbers of SNVs vary between strains from 34, in the reference strain JF4335, to 8520, within the strain JF4495 from the United Kingdom. Out of the remaining 34 variants in the reference strain JF4335 only 13 were not flagged as low quality by the Samtools filtering and are either short INDELs or SNVs with an unclear resolution that were therefore not corrected after the assembly of the reference sequence with PacBio. The number of SNVs for each strain is given in **Table 1**. Using the determined SNVs of all strains we created an alignment and built a more detailed phylogenetic tree based on the generalized time reversible substitution model in RAxML. In the resulting tree (**Figure 4A**) we can confirm the clustering of the strains isolated in the New Zealand, Australia and the United Kingdom which is set apart from the remaining strains isolated from Brazil, South Africa, United States, Switzerland and Sweden, independent on the year of isolation. However, since the differences were too small between several strains they appear grouped on the SNV based evolutionary tree. The cladogram shown in **Figure 4B** allows the visualization of the separation of each strain.

### CRISPR Sequences

**FIGURE 5:**
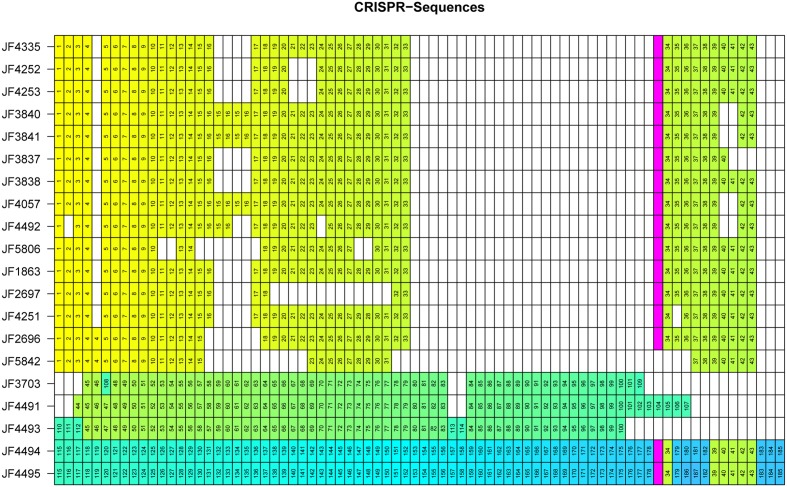
Matrix representation of CRISPR spacers for each individual *C. chauvoei* strain. Identical spacers are assigned the same numerical value and color. The IS*256* transposase is indicated with in pink and divides the CRISPR sequences into two arrays. White spaces are artificial due to the visual representation lining up identical CRISPR spacer sequences and do not represent DNA sequences of the individual genomes. The order of the strains, from top to bottom was arranged to follow clusters and subgroups of the phylogenetic tree of the SNV analysis (**Figure 3**).

The CRISPR sequences found with CRISPRFinder (Grissa et al., 2007) were analyzed for overlapping CRISPR sequences within the genomes of the individual strains and CRISPR sequences among the 20 strains analyzed. We found between 30 and 77 CRISPR sequences per strain in our collection. All CRISPR sequences contain identical interspaced direct repeats (DR) of 30bp length (5′-GATTAACATTAACATGAGATGTATTTAAAT-3′). In total, we discovered 1043 CRISPR spacer sequences in the strains analyzed. To individualize spacer sequences within our complete strain collection and identical spacer sequences between the individual strains, we applied an all-vs.-all alignment using NUCmer and an in-house R script to sort them into groups. After both these steps, we got 187 unique spacer sequences and numbered them individually (**Figure 5**). All spacer sequences are represented in 5′ to 3′ order of appearance downstream of *cas2* in the respective genome (**Figure 5**). For reasons of visual representation, we aligned identical CRISPRs underneath each other wherever possible. The resulting analysis shows a clustering of the *C. chauvoei* strains into a European/African/American cluster and an Australia/New Zealand/United Kingdom cluster. This clustering corresponds to that found in the comparison taking into account the four virulence genes *cctA*, *nagI*, *nagH* and *nanA* or the whole genome SNV analysis, thus confirming the phylogenetic clustering that we found with the two latter approaches.

The spacer sequence analysis of the CRISPR locus of the various *C. chauvoei* strains reveals that strains belonging to the Australia/New Zealand/United Kingdom cluster strongly and differ from all other strains since they share barely any of the spacer sequences with the strains from the cluster Africa/Europe/America. Furthermore, CRISPR analysis of the Australia/New Zealand/United Kingdom cluster reveals a clear subdivision leading to subgroups A (containing a strain from Australia and two from New Zealand) and subgroup B (a strain from each New Zealand and United Kingdom), which are distinctly different from each other and share no spacers among each other (**Figure 5**).

A striking feature found in the CRISPR locus of most strains, particularly the strains for the Africa/Europe/America cluster is the presence of a transposase belonging the IS*256* family of insertion sequence (IS) in the middle of the repeats splitting the CRISPRs into two arrays (**Figure 5**; pink box). This transposase is present in all but one strain of the Africa/Europe/American cluster and in subgroup B (New Zealand/United Kingdom) but is not present in subgroup A of the Australia/New Zealand strains. The sequence identity between the IS*256* transposase between different strains is ≥ 99%. Surprisingly this transposase is lacking in a recent strain from Brazil (JF5842) that was isolated in 2015, hence later than the other Brazilian strains, which do contain this transposase. Interestingly, in strain JF5842 not only the IS*256* transposase, but also the CRISPR spacers #32 and #33 upstream and #34, #35 and #36 downstream of the transposase are lacking in comparison to the closest relative strain JF2696 (Brazil 2002). Furthermore, the presence of the IS*256* transposase within the African/European/American cluster and in subgroup B of the New Zealand/United Kingdom strains is particularly interesting as it is lacking in subgroup A of this latter cluster. Moreover, the transposase in the New Zealand and United Kingdom strains is followed at its 3′-end by spacer element #34 that is common to all the strains of the African/European/American cluster except the recent Brazilian strain discussed above.

Overall, the analysis of the spacer sequences of the CRISPR locus among strains allows a very clear differentiation of most individual strains of *C. chauvoei* and estimate their recent parentage, which by other means such as SNV analysis result in much less resolution power due to the particularly high genetic homology of *C. chauvoei* strains both over space and time. Eighteen isolates are from bovine, three from ovine and one without known animal species. They do, however, not cluster according to the animal species isolated from independently if clustering as done by CRISPR typing or SNV based grouping.

### Comparison of Flagellin Genes in Different Strain Groups

The reference strain JF4335 contains a prominent cluster of three putative genes *fliC1*, *fliC2* and *fliC3*, in consecutive order, encoding three different paralog alleles of the structural flagellin proteins. Based on nucleotide-derived a.a. sequences the three flagellin paralogs show 91.8% a.a. identity among each other. The read mapping of strains revealed that in particular the strains from subgroup B (JF4494 New Zealand and JF4495 United Kingdom) show very low coverage in the read mapping in the region of the structural flagellin genes indicating that these strains possess fewer alleles of flagellin genes.

To further investigate the flagellin genes in several strains, the reference flagellin genes plus the flanking genes were mapped to the contigs of each strain. The genes flanking the *fliC1, fliC2, fliC3* locus were annotated as the flagellar cap protein D, gene *fliD*, plus a short hypothetical protein upstream and gene *fliB*, encoding a putative lysine-N-methylase downstream of *fliC3* in the reference strain JF4335. As expected, mapping of the contigs primarily revealed a difference in copy number of the structural flagellin genes between *fliD* and *fliB* or homologs of them. Strains JF4494 (New Zealand) and JF4495 (United Kingdom) that form subgroup B, both only show a single copy of a structural flagellin gene referred to as *fliC1* which results in 86 to 87% identical a.a. residues compared to FliC1, FliC2, FliC3 of reference strain JF4335. This difference of copies of *fliC* alleles was confirmed by PCR analysis (data not shown) using primers FLIDL (5′ -CTAAGCTTGAAGTTGCAATGAAC- 3′) and FLIBR (5′ -GAATGTGAAGATCATTGCTGTAA- 3′), which resulted in a fragment of 1.7 kb for strain JF4494 with one copy and a fragment of 5 kb for strains with 3 copies of *fliC* alleles, such as the reference strain JF4335. The Swedish strain JF4252 and the United States strain JF4492 show two copies of structural flagellin genes. In JF4252 (Sweden) one gene showed full homology to the FliC2 and the other 99.8% identical a.a. to FliC1 of JF4335. In a multi-sequence alignment on the ORFs, they have a 92.1% pairwise nucleotides identity and a 90.3% pairwise a.a. identity among each other. Similar results were found for the United States strain JF4492 that has one of its structural flagellin genes homologous to FliC3 and one with 96.1% a.a. identity to FliC1 of reference strain JF4335. A complete table of all nucleotide and a.a. similarities between the various structural flagellin genes is given in **Table 2**.

**Table 2:** Pair-wise identities of all the FliC genes in the strains JF4335, JF4252, JF4492, JF4494, and JF4495 created by a MAFFT multi-sequence alignment.

Nucleotide	JF4335 fliC1	JF4335 fliC2	JF4335 fliC3	JF4252 fliC1	JF4252 fliC2	JF4492 fliC1	JF4492 fliC2	JF4494 fliC1	JF4495 fliC1
JF4335 fliC1	1	0.931	0.931	0.928	0.997	0.931	0.964	0.866	0.866
JF4335 fliC2		1	0.969	0.997	0.934	0.969	0.967	0.868	0.868
JF4335 fliC3			1	0.972	0.934	1	0.936	0.861	0.861
JF4252 fliC1				1	0.931	0.972	0.964	0.87	0.87
JF4252 fliC2					1	0.934	0.967	0.864	0.864
JF4492 fliC1						1	0.936	0.861	0.861
JF4492 fliC2							1	0.872	0.872
JF4494 fliC1								1	1
JF4495 fliC1									1

**Protein**	**JF4335 FliC1**	**JF4335 FliC2**	**JF4335 FliC3**	**JF4252 FliC1**	**JF4252 FliC2**	**JF4492 FliC1**	**JF4492 FliC2**	**JF4494 FliC1**	**JF4495 FliC1**

JF4335 FliC1	1	0.918	0.918	0.918	0.998	0.918	0.961	0.826	0.826
JF4335 FliC2		1	0.966	1	0.92	0.966	0.956	0.835	0.835
JF4335 FliC3			1	0.966	0.92	1	0.923	0.823	0.823
JF4252 FliC1				1	0.92	0.966	0.956	0.835	0.835
JF4252 FliC2					1	0.92	0.964	0.823	0.823
JF4492 FliC1						1	0.923	0.823	0.823
JF4492 FliC2							1	0.838	0.838
JF4494 FliC1								1	1
JF4495 FliC1									1


**FIGURE 6:**
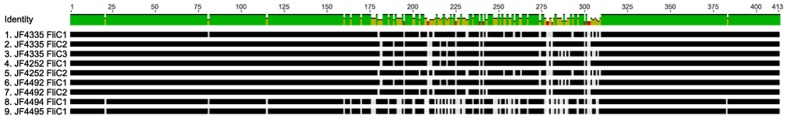
Multi-sequence alignment (MSA), visualized in Geneious v10.1.2, of the ORFs, coding for Flagellin genes FliC, of strains JF4335, JF4252, JF4492, JF4494, and JF4495 as representatives of the detected difference in copy numbers in the strains. The MSA reveals that the N- and C- termini are mostly conserved, while the variable domain is in the middle of the FliC.

Interestingly, the 413 amino-acid long sequences of FliC1, FliC2 and FliC3 among themselves and among the various strains analyzed show their variability between position 174 and 302, whereas the N- and C-termini are mostly conserved (**Figure 6**). We detect a putative conserved domain described as flagellin D0/D1 domain (IPR001029) in all structural flagellin genes in all strains analyzed. This can lead to the assumption that they fulfill a similar task in all strains. However, this central hyper variable region could be of critical importance when designing recombinant vaccines using flagellin proteins.

## Discussion

Blackleg is a global, severe, and mostly lethal bacterial infectious disease of ruminants caused by the sporulating bacterium *C. chauvoei*. Due to its rapid progression, there are hardly any therapies for the affected individuals. In contrast, preventive measures by vaccination with empirically developed bacterin vaccines are efficient and protect animals grazing on meadows that are contaminated by spores of *C. chauvoei*. In spite of the worldwide spread of blackleg, there are hardly any studies published on the molecular mechanisms of pathogenicity and epidemiological spread of the organism. These, however, would be of high importance in the design and composition of vaccines and testing their efficacies. Interestingly, international bacterial strain collections conserve hardly any clinical strains of *C. chauvoei*, besides the type strain whose exact origin and date of isolation is unknown. We therefore performed full genome sequencing and bioinformatics analysis of a collection of 20 strains of *C. chauvoei* isolated from bovine and ovine sources over the last 64 years on four different continents that were maintained at the internal strain collection of the Institute of Veterinary Bacteriology, University of Bern. The Swiss strains were isolated in the past from clinical cases of blackleg in cattle. The other strains were either purchased from international strain collections or obtained from other laboratories for purpose of species identification. In spite of vast temporal and geographical diversity of these strains, the phylogenetic analysis based on 37 OTU-free marker gene families of the different *C. chauvoei* strains showed virtually no genetic variations and behaved like a single strain. In addition, the analysis of the major virulence genes such as the main cytotoxin CctA, the sialidase NanA and the two hyaluronidases NagI, NagH show hardly any variations on the a.a. level indicating identical phenotypes. Hence, the evolution of *C. chauvoei* seems to have stopped from the point of view of metabolism and virulence factors and the pathogen seems to be well adapted to its bimodal way of life using as host, cattle and sheep, where it replicates and the environment, the meadows, where it persists as spores.

While SNVs, which mostly are located in intergenic segments of the chromosome, allow a finer differentiation of the strains, the spread of the pathogen can be followed by the large number of CRISPR elements. While the CRISPR associated protein genes in all strains are well conserved, we detected in total 187 unique spacer sequences within the CRISPR elements that are reminiscences of successfully warded phage attacks. These show significant differences between the strains from Africa, continental Europe and Northern and Southern America and strains from United Kingdom, Australia and New Zealand. There are surprisingly very little differences found between strains from Switzerland, Sweden, Brazil and South Africa, which contain virtually identical spacer sequences throughout the 40 different CRISPR elements. The presence of an insertion element from the IS*256* family, inserted after approximately the 30th spacer element is peculiar. In a recently isolated strain from Brazil from 2015, this insertion element is deleted and, in comparison with the two strains isolated in 2002 the excision of the IS seems to have removed also five flanking CRISPRs. This indicates that this IS element is still active and able to transpose.

The strains from Australia, New Zealand and the United Kingdom reveal totally different spacer sequences, compared to the strains from Switzerland, Sweden, Brazil and South Africa, which reflect the close cultural and agricultural exchange between these countries over a long historical period. This is illustrated with two strains JF4494 isolated in 1963 in New Zealand, which is identical to strain JF4495 isolated 10 years previously in the United Kingdom. The fact that there are two completely different CRISPR sequences found in strains in New Zealand indicates that the pathogen must have been introduced twice in the past, once in connection with Australia and one in connection with the United Kingdom. Interestingly, the strains from the second event (JF4494 and JF4495) not only are identical with regard to the CRISPRs but also show the presence of the IS*256* directly followed by CRISPR spacer #34 which is found in the Africa/Europe/America cluster. Furthermore, both strains also contain five CRISPR spacers (#39 – #43), probably as recently acquirements indicating that they had to fend off the same or similar phages as the strains from the Africa/Europe/America cluster.

Since flagella have been considered as important protective antigens, we have put particular attention to the genetic variations of flagellar genes among the strains analyzed. While the basal flagellar genes are mostly identical or show only irrelevant differences among all strains, we detected differences in the flagellin genes *fliC*. Most importantly, *fliC* appears as three allelic copies with 91.8% identity in most strains, while two strains, one from New Zealand and one from the United Kingdom, only contained a single copy. A strain from Sweden and the one from United States also contained two copies. Since the three alleles of *fliC* are contiguous and very similar among each other, the loss of one or two alleles can be explained by homologous recombination events. Furthermore it has to be noted that a single *fliC* allele in the relatively ancient strain from New Zealand and from the United Kingdom show only in 86% to 87% identity to the *fliC* alleles that are present in most other strains. If the FliC antigens do play a role in protective immunity induced by vaccines, an assessment of the *fliC* alleles in currently circulating strains in the areas where vaccines are used would be useful. The current data, in particular the PCR primers developed in this study will provide a useful tool for this purpose.

While the study contains relatively few strains taken from most distant geographical places itn has to be taken into consideration that the data need to be interpreted with precaution with regards to specific countries or animal origins of the strains. It is interesting to note that the few ovine strains included in this study do not correlate to any of the grouping made by CRISPR, SNV or virulence gene typing. Hence, the clusters of *C. chauvoei* described seem not to show host predilection.

Besides the relatively minor genetic differences found in the flagellar genes and in the CRISPR spacers our analysis reveals that the genome of *C. chauvoei*, which is the smallest among the species of the genus *Clostridium*, is highly conserved among strains collected from most distant geographical areas and over a long time frame. This indicates that *C. chauvoei* is a highly specialized and successful pathogen whose evolution has reached a dead end.

## Author Contributions

JF, LF, and SD conceived the study and performed the genomic analyses. PN performed the strain isolations and DNA preparations. Bioinformatic analyses were made by SI-A and LR. PC established the phylogenic relationship using by PhyloSift. RZ and AdV provided the strains and metadata from strains of Brazil.

## Conflict of Interest Statement

The authors declare that the research was conducted in the absence of any commercial or financial relationships that could be construed as a potential conflict of interest. The research was done with the final aim to replace the currently used guinea pig potency test for batch release of blackleg vaccines by an *in vitro* procedure thus avoiding challenge of animals with pathogens.

## Footnotes

1 Bergey CM (2012). vcf-tab-to-fasta; http://code.google.com/p/vcf-tab-to-fasta

2 Rambaut A.; http://tree.bio.ed.ac.uk/software/figtree/

3 Rambaut A.; http://tree.bio.ed.ac.uk/software/figtree/
