# Corrigendum: *Clostridium chauvoei*, an Evolutionary Dead-End Pathogen

**DOI:** 10.3389/fmicb.2018.00421

**Published:** 2018-03-06

**Authors:** Lorenz Rychener, Saria In-Albon, Steven P. Djordjevic, Piklu Roy Chowdhury, Pamela Nicholson, Rosangela E. Ziech, Agueda C. de Vargas, Joachim Frey, Laurent Falquet

**Affiliations:** ^1^Institute of Veterinary Bacteriology, Vetsuisse Faculty, University of Bern, Bern, Switzerland; ^2^The iThree Institute, University of Technology Sydney, Ultimo, NSW, Australia; ^3^Department of Preventive Veterinary Medicine, Federal University of Santa Maria, Santa Maria, Brazil; ^4^Department of Biology, Swiss Institute of Bioinformatics, University of Fribourg, Fribourg, Switzerland

**Keywords:** *Clostridium chauvoei*, CRISPR, virulence genes, flagellin genes, blackleg, dead-end evolution

In the published article, Pamela Nicholson was not included as an author. In addition, author names were incorrectly spelled as Saria InAlbon and Piklu R. Chowdhury. The correct spellings are Saria In-Albon and Piklu Roy Chowdhury.

The authors apologize for these errors and state that they do not affect the scientific conclusions of the article in any way.

The original article has been updated.

## Author contributions

JF, LF, and SD conceived the study and performed the genomic analyses. PN performed strain isolations and DNA preparations. Bioinformatic analyses was made by SI-A and LR. PC established the phylogenic relationship using by PhyloSift. RZ and AdV provided strains and metadata from strains of Brazil.

### Conflict of interest statement

The authors declare that the research was conducted in the absence of any commercial or financial relationships that could be construed as a potential conflict of interest.

